# Installation of speed humps and pedestrian-motor vehicle collisions in Toronto, Canada: a quasi-experimental study

**DOI:** 10.1186/s12889-015-2116-4

**Published:** 2015-08-12

**Authors:** Linda Rothman, Alison Macpherson, Ron Buliung, Colin Macarthur, Teresa To, Kristian Larsen, Andrew Howard

**Affiliations:** Child Health Evaluative Sciences, The Hospital for Sick Children, 555 University Ave., Toronto, ON Canada M5G 1E2; Faculty of Health-School of Kinesiology & Health Science, York University, Norman Bethune College, 4700 Keele St., Room 339, Toronto, ON Canada M3J 1P3; Department of Geography, University of Toronto Mississauga, 3359 Mississauga Road N, South Building, Mississauga, ON Canada L5L 1C6; Research Institute, The Hospital for Sick Children, 555 University Ave., Toronto, ON Canada M5G 1E2; Orthopaedic Surgery, The Hospital for Sick Children, 555 University Ave., Toronto, ON Canada M5G 1E2

## Abstract

**Background:**

Evidence related to the effectiveness of speed humps on reducing pedestrian-motor vehicle collisions (PMVC) has been conflicting. The purpose of this study was to determine the association between speed hump installation and changes in PMVC rates in Toronto, Canada.

**Methods:**

Speed humps were mapped along with police-reported pedestrian collisions from 2000–2011 and built environment roadway characteristics. A quasi-experimental study identified collision counts before and after speed hump installation, modeled using repeated measures Poisson regression adjusted for season and roadway characteristics. Stratified analyses were conducted by age group and injury severity.

**Results:**

There were 27,827 PMVC, with 1344 collisions along 409 roadways with speed humps. PMVC incidence rates/meters of road/month decreased after installation of speed humps (IRR 0.78 95 % CI 0.66, 0.91). Winter, collector roads (versus local), pre-amalgamated city centre and increased land use mix were associated with more collisions. The association between speed humps and PMVC rates decreased more for children (IRR 0.57, 95 % CI 0.41, 0.79) than for adults (IRR 0.80, 95 % CI 0.68, 0.95).

**Conclusions:**

Speed humps are an easily replicated method of traffic calming which have a significant protective effect on PMVC on the roadways where they are installed, particularly for children. There is a need for an area-wide analysis to determine the effects of the installation of speed humps to ensure that PMVC are not being displaced to surrounding roadways.

## **Background**

Road traffic crashes were responsible for up to 50 million non-fatal injuries worldwide and another 1.24 million fatalities in 2010 [1]. Road injury ranked seventeenth for Years Lived with Disability (YLDs) and ranked eighth for global annual death rates with a 30 % increase and a 47 % increase respectively since 1990 [2]. Road injury is predicted to rise to the fifth leading cause of death globally and the seventh leading cause of Disability Adjusted Life Years (DALYs) lost by 2030 [3–5].

Speed is the major risk factor for motor vehicle crashes, and directly influences injury severity [6, 7]. Pedestrians have less than a 50 % chance of surviving a collision at ≥45 km/h, but a 90 % chance of surviving collisions at ≤ 30 km/h [6, 8]. The likelihood of death for car occupants is 20 times higher at an impact speed of 80 km/h, than it would be at an impact speed of 32 km/h [9]. Therefore, much of the focus of the prevention of motor vehicle collisions is on speed reduction.

Traffic calming strategies generally refer to physical changes to the roadway that are designed to reduce speed in urban areas. Traffic calming strategies have been classified as 1) physical vertical and horizontal shifts in traffic (e.g. speed bumps and humps, raised crosswalks) 2) optical measures (e.g. road surface treatment) 3) redistribution of traffic (e.g. one-way streets) and 4) changes to the road environment (e.g. vegetation) [10]. Speed humps are one of the most common physical traffic calming interventions used in urban areas. They are characterized by a gradual raised area in the pavement surface as opposed to speed bumps, which are composed of an abrupt raised area which are more jarring to motorists and are not recommended for use on public roads. Speed humps have been demonstrated to effectively reduce vehicle speeds [11–13] and have been associated with the reduction of motor vehicle collisions [14–16].

Evidence on the effect of speed humps and pedestrian-motor vehicle collisions (PMVC) however, has been conflicting [17]. A systematic review of before-after controlled studies suggested that area-wide traffic calming reduced traffic injuries and deaths in urban areas; however, the pooled incidence rate ratios of the 14 studies specific to PMVC was non-significant (1.1, 95 % CI 0.88, 1.16) [10]. More recently, a cross-sectional study of child PMVC and walking to school in Toronto, Canada found higher densities of traffic calming in areas near schools consisting mostly of speed humps, were associated with higher incidence rates of motor vehicle collisions involving child pedestrians [18]. Although it may be assumed that by decreasing vehicle speeds, PMVC incidence rates will also decrease, this has not definitively been established through empirical evaluation [19]. This study used a quasi-experimental pre-post design to determine whether the installation of speed humps were associated with a change in PMVC incidence rates in the City of Toronto, Canada.

## Methods

A quasi-experimental design was used to compare incidence rates of PMVC before and after installation of speed humps. Roadways were included where humps were installed during the study period (January 2000–December 2011). Collision data from 2000–2011 were extracted from MVC police reports filed and verified by the City of Toronto, Transportation Services Division and included longitudinal and latitudinal geographic coordinates, age and injury severity.

### Outcome

The outcome was the incidence rate of PMVC. Police-reported PMVC were mapped onto Toronto centre line shapefiles provided by the City of Toronto open data site, using ArcGIS, ArcMap version 10. A 25 m buffer, representing the width of the roadway, was created to capture all pedestrian collisions that occurred close to or on the road.

Traffic calming roadway segment shapefiles were provided by the City of Toronto, Transportation Services Division. The analysis was restricted to speed humps, which represented the majority of traffic calming features in the City of Toronto (97 %). Speed hump segments constructed along the same roadway were combined and mapped onto City of Toronto street centre line data. Collisions were assigned to a roadway with speed humps and were excluded if 1) the collision occurred beyond the 25-meteres roadway width 2) occurred on the same day of speed hump installation.

### Primary exposure

Speed humps were installed at various times throughout the 12 year study, with varying pre and post-installation time periods assigned.

### Covariates

Season was included as a covariate and was dichotomized into summer (April–September) or fall-winter (October–March). Built environment roadway characteristics were measured along the roadway with speed humps and also included as covariates. Road type data was provided by the City of Toronto and was included as a binary variable which identified collector and local roads with speed humps. A binary variable identified the neighbourhood; older pre-amalgamated City of Toronto and the newer inner suburbs. Metropolitan Toronto was amalgamated in 1998 with 5 inner ring suburban municipalities. This amalgamation formed a city with an older central urban core with many pre-World War II neighbourhoods characterized by straighter and more connected gridded street patterns, surrounded by newer inner suburbs with a curvilinear less connected street network and more cul-de sac’s typical of many post-WWII suburban areas [20]. Parcel level data from the Municipal Property Assessment Corporation (MPAC) was used to formulate a land use mix variable. Finally, land use mix along the frontage of the roadway with the speed humps was measured using an entropy index where scores of 0 = single land use, 1 = equal distribution of all land use classifications, i.e. residential, commercial, institutional, industrial, recreational/open space) [21].

#### Statistical analysis

Statistical analysis was conducted using SAS V.9.3 and STATA V.12.1. The collision occurrence was measured using incidence density/roadway meter/month, which was the unit of analysis. This rate reflected the number of collisions per each meter of roadway that had speed humps installed per month over the 12 year period. The pre-installation period without the speed humps was designated as the reference value, with the intervention period after hump installation.

The association between the PMVC count per speed hump roadway/meter/month adjusted for season, road type, neighbourhood, and land use mix was assessed using repeated-measures Poisson regression analysis. Generalised estimating equations (GEE) with an autoregressive correlation structure were used to fit the models. The rate denominator was the number of meters per month for each speed hump roadway. The dichotomous exposure variable indicated whether the collision occurred pre or post installation. Incident rate ratios (IRR) with 95 % confidence intervals were calculated. The overall percent reduction in PMVC counts by year on roadways with no speed hump installation were examined over the 12 year period and compared with the percent reduction in PMVC on roadways with speed hump installation.

The total number of PMVC prevented by installation of speed humps was calculated by multiplying the preventive fraction of installation (1- incident rate ratio) by the total number of PMVC along the roadways where speed humps were installed from 2000–2011. As most speed humps are installed on local residential roads in Toronto [22], the potential number of PMVC avoided if speed humps had been installed on all local roadways was also calculated by multiplying the preventive fraction of speed hump installation *only on local roadways* by the total number of PMVC that occurred on local roads over the 12 year period.

Stratified sub-analyses were conducted by age and injury severity using models adjusted for the same covariates; season, road type, neighbourhood and land use mix. Age was categorized as children and youth (ages 0– 14 years), adults (15–59) and older adults (ages ≥60 years) [2]. Injury severity was categorized based on Toronto Police Service injury classification: no injury; minimal injury (no medical attention); minor injury (visit to the emergency department (ED); major injury (hospital admission); and fatal injury. Collisions resulting in no injuries and minimal injuries were combined. As there were few fatal injuries, major and fatal injuries were also combined.

## Results

There were 1463 roadway segments with speed hump construction dates from 2000–2011. Roadway segments with speed humps were combined into 409 total roadways, with two roadways removed, as humps were installed January 1, 2000, which allowed for no pre-installation time for the analysis. Three roadways were removed as they were laneways which are not classified in the City of Toronto’s roadway classification system [23]. Therefore, the total number of classified roadways with speed humps in the analysis was 404 and 176 total kilometers.

There were 27,827 reported PMVC from 2000–2011; 1344 of these collisions occurred on a roadway with speed humps which had construction dates after January 1, 2000. One collision occurred on the same day of construction, which was excluded.

There were 23,241 speed hump months prior to the installation of speed humps with an unadjusted average rate of 0.067 collisions/km/month, and 35,339 post installation months following installation with an unadjusted average rate of 0.060 collisions/km/month. More than half of PMVC occurred during the winter (748, 56 %, Table 1). The majority of the speed humps were installed on local roads (344, 84.1 %) and were located in the pre-amalgamated city (245, 60.6 %). The average entropy index land use score was 0.26 indicating a low level of land use mix on roadways with traffic calming. Eighty-two percent of the roadways with speed humps were in residential areas.

**Table 1:** Frequency and adjusted incident rate ratios of pedestrian-motor vehicle collisions with 95 % confidence intervals by speed hump installation (pre, post), season and roadway characteristics

	Number (%)	Adjusted IRR
		(95 % CI)
Outcome:		
Collisions	1344	n/a
Primary exposure:		
Speed hump implementation		
Pre implementation	594 (44.2 %)	1.00
Post implementation	750 (55.8 %)	0.78 (0.66, 0.91)
Covariates:		
Season (month of collision):		
Non-winter	596 (44.3 %)	1.00
Winter	748 (55.7 %)	1.26 (1.12, 1.42)
Roadway characteristics (n = 404):		
Road type:		
Local	344 (84.1 %)	1.00
Collector	60 (14.7 %)	1.56 (1.18 ,2.08)
Neighbourhood:		
Inner suburbs	159 (39.4 %)	1.00
Pre-amalgamated City of Toronto	245 (60.6 %)	1.63 (1.23, 2.14)
Land use mix:		
Mean entropy score	0.26 (SD ± 0.21)	2.48 (1.42, 4.34)

The Poisson regression model resulted in a PMVC incidence rate ratio of 0.78 (95 % CI 0.66, 0.91) (Table 1) after speed hump installation, while controlling for season and built environment roadway characteristics. This represented a preventive fraction of 22 % and a reduction of 296 PMVC after installation. There was a positive association of PMVC with winter months (IRR = 1.26, 95 % OR 1.12, 1.42). Collector roads with traffic calming, were associated with higher PMVC incidence rates compared to local roads (IRR = 1.56, 95 % CI 1.18, 2.08). Traffic calming in older preamalgamated city neighbourhoods, versus inner suburbs (IRR = 1.63, 95 % CI 1.23, 2.14) and roadways with greater land mix (IRR = 2.48, 95 % CI 1.42, 4.34) were associated more PMVC. The presence of over-dispersion was tested by also modelling the outcome using negative binomial regression. The estimates using Poisson and negative binomial modelling were almost identical; however, the Poisson model had a better fit, as indicated by the QIC (Quasi-Akaike Information Criterion) goodness of fit statistic for GEE models.

The reduction in PMVC incidence rates from 2000–2012 associated with the installation of the speed humps was greater than what occurred on roadways where there were no speed humps installed over the study period. Collisions on roadways which had no speed humps installed had 2386 reported collisions in 2000, which dropped to 2172 in 2011. This 9 % reduction over the 12 years was substantially less that the 22 % reduction associated with the installation of the speed humps.

Age was missing for 51 collisions, and these were excluded from the age stratified analyses. The majority of PMVC occurred in adults, ages 15–59 (Table 2). Over half of collisions resulted in an emergency department visit or hospital admission. There were 9 adult fatalities during this time period at speed hump locations (4.3/1000 km/year) compared to 337 adult fatalities (4.9/1000 km/year) in the rest of the city.

**Table 2:** Incident rate ratios of pedestrian-motor vehicle collisions stratified by age and injury severity post-installation of speed humps

Collision	# of Collisions	IRR of PMVC
	(*n* = 1344)	(95 % CI) Adjusted for covariates^a^
Age		
Child (0–14 years)	190 (14.1 %)	0.56(0.40, 0.79)
Adult (15–59 years)	843 (62.7 %)	0.80 (0.67, 0.95)
Older adults (60+)	260 (19.4 %)	0.83 (0.63, 1.09)
Missing	51 (0.4 %)	-
Injury Severity		
No injury/minor	607 (45.2 %)	0.78 (0.63, 0.96)
Minimal (visit to ED)	627 (46.7 %)	0.80 (0.66, 0.96)
Severe/Fatal	110 (8.2 %)	0.66 (0.44, 1.01)

^**a**^Adjusted for season, road type, neighbourhood, and land use mix

In PMVC involving children, there was a 44 % reduction of the adjusted collision incidence rate after speed hump installation (IRR = 0.56, 95 % CI 0.40, 0.79, Table 2). Winter, being in the pre-amalgamated city centre and collector roads were not associated with collision rates in children; however, mixed land use (IRR = 5.59, 95 % CI 2.34, 13.38) was a significant positive correlate of PMVC after controlling for speed hump installation. In adults, installation was associated with a 20 % reduction in PMVC incidence rates (IRR = 0.80, 95 % CI 0.67, 0.95). There was a 22 % reduction in PMVC incidence rates involving no/minor injuries (IRR = 0.78, 95 % CI 0.63, 0.96), and a 20 % reduction in minimal injuries requiring a visit to the emergency department (IRR = 0.80, 95 % CI, 0.66, 0.96). Although there were similar effect sizes for older adults and severe/fatal PMVC to the total sample of all ages, these were not statistically significant likely due to the small number of PMVC.

There was a 26 % reduction in PMVC incidence rates (0.74, 95 % CI 0.62, 0.89) on local roads after speed hump installation after controlling for winter and built environment roadway characteristics. Of the 27, 827 total number of PMVC that occurred from 2000–2011, 2119 (8 %) occurred on local roads. Speed hump installation on all city local roads could potentially have avoided 552 PMVC over a 12 year period, or 46 PMVC/year.

## Discussion

The installation of speed humps was associated with a 22 % reduction of PMVC incidence rates overall and with a 26 % reduction specifically on local roads. This protective effect was seen across all ages, but was especially strong in children and youth ages 0–15, where there was an associated 43 % reduction of PMVC incidence rates. “The effect of speed hump installation was substantially greater than the decreasing trend in collisions over the 12 year time period.

Speed humps are a roadway modification directed solely at changing driver behaviour, which have been assumed to decrease PMVC by decreasing traffic speed. However, the effect of speed humps on pedestrian behaviour, particularly children, is unknown [19]. The results of these analyses suggest that the installation of speed humps has the expected effect; reducing PMVC incidence rates, even after controlling for a variety of built environment roadway characteristics.

The effectiveness of speed humps found in this study was similar to those reported previously. A study reported a 53–60 % reduction in the odds of injury and death among children struck by an automobile in their neighbourhood with area-wide traffic calming [15]. A Cochrane method review including 14 controlled-before-after studies investigating area-wide general traffic calming and pedestrian collision outcomes, indicated no effect of traffic calming with a pooled rate ratio of 1.01 (95 % CI 0.88, 1.16). The review emphasized heterogeneity between studies, and the importance of proper evaluation of well-designed controlled studies [10]. Considering the larger effect size of speed humps on children compared to adults in the present study, it may be appropriate to specifically focus future studies on the effects of traffic calming on children, especially considering the higher burden of child pedestrian injury on smaller local streets where speed humps are generally installed.

There is some controversy regarding the use of speed humps. They are seen to be problematic by some as they delay both emergency response public transit vehicles, and as a result, they are not installed on emergency response or transit routes in Toronto [22]. They also have the potential for increasing traffic noise and pollution (i.e., emissions and brake dust produced during deceleration/acceleration), they may divert traffic onto other residential streets without speed humps, and they could produce discomfort for some vehicle occupants [22, 24]. There has been some recent media attention in Toronto, claiming that speed humps have been overused [25]. The process of obtaining speed humps can frequently be lengthy and onerous, as it necessitates public support and political will. However, there are definite advantages of speed humps, in that they are relatively inexpensive compared to other roadway modifications, easily replicable, and there is strong evidence that they are effective in slowing vehicles down [11–13, 24]. Road type, pre-amalgamated city (versus inner suburbs), mixed land use and winter season were positively associated with PMVC in adults which are risk factors for that have been previously identified [26–30]. In children however, only road type and mixed land use were associated with PMVC along specific roadways after controlling for traffic calming. Positive associations between PMVC in children and roads with higher traffic volumes and speed have been found previously [31]. Evidence regarding the association between mixed land use and child PMVC is inconsistent. In a previous study using the same collision data, mixed land used was not significantly associated with child PMVC [18]. However, both the collisions and the built environment roadway characteristics were measured using much larger areal level school attendance boundaries. Other studies have found either no relationship between PMVC and mixed land use, using census block areas [32], or a positive relationship, again using school catchment areas [33]. These discrepancies may be the result of different methods of measurement of mixed land use, or may indicate issues related to the modifiable areal unit problem (MAUP), where differences in how space is partitioned affect the results [34].

One of the limitations studies of traffic interventions is the lack of exposure data related to both pedestrians and motor vehicles. Pedestrian exposure and traffic volume could not be accounted for in this study, as there are no regularly collected counts on local streets in Toronto. It might be expected however, that pedestrian volumes increased on local streets where there were speed humps installed due to the lower speed traffic, with potentially decreased traffic volumes as drivers may choose to drive on streets without speed humps. Regardless of how the calming affected exposure to traffic, the desired effect has been achieved; that is, to reduce PMVC along a roadway which has been identified by the City of Toronto to have dangerous excessive speed. It is however, important to consider the possibility that the speed humps have displaced traffic and also potentially, PMVC to neighbouring streets. Further work is necessary, therefore, to examine the spatial patterns of PMVC before and after the installation of speed humps on streets surrounding calmed roadways.

Another potential limitation of the pre-post study quasi-experimental design is related to regression to the mean effects, where extreme values of the injury outcome would regress towards the mean with multiple testing [35]. Therefore, a roadway with high occurrence of injury may have a reduction, merely because their pre-installation rates were extreme. This phenomena is not likely an issue here as roadways are not selected for speed hump installation in Toronto in response to high collision counts. Rather, the process is related to criteria including: community support, safety indicators (including presence of sidewalks, impact on emergency vehicles), and technical criteria (i.e. speed, traffic volume, block length and public transit) [22]. Other measures to increase road safety were not considered within these analyses; therefore, if the implementation of speed humps were part of a broad risk reduction strategy, the speed humps could potentially act as a confounding variable for another traffic control measure. Finally, there are limitations of using police-reported collision data, as less severe collisions and child pedestrian-motor vehicle collisions tend to be underreported [36, 37]. Underreporting would not likely have affected the results of this study, as there is no reason to believe that reporting may have systematically changed before and after speed hump installation.

The strengths of the study included the pre-post design, which controlled for temporal, seasonal and built environment roadway characteristics. Multivariable modeling was used to test the association between the installation of speed humps and PMVC while controlling for some features of the roadway built environment. The repeated measures design, also controlled for other potentially confounding factors related to the built environment around the speed humps, which were not considered in the model. Finally, as speed humps are applied universally on local residential roads, with similar speed and volume criteria as described by specific guidelines, the results could be generalized to most urban settings in high and middle income countries [38].

## Implications and conclusions

In Toronto, speed humps were found to be effective in reducing PMVC incidence rates along the roadways where they have been installed. The greatest positive effect of speed humps was observed in child pedestrians, as speed humps are generally located on local roadways in residential areas in Toronto where children tend to walk. More speed hump installation on local roads has the potential to further decrease PMVC; however, there is some controversy regarding their overuse, cost, and the duration of the application approval and installation process. Other types of traffic calming have been proposed on higher speed roads in mixed land use areas, such as medians and road narrowing [39–41]. Although the effectiveness of speed humps on reducing PMVC rates along the roadways where they are installed has been established, there is a need for an area-wide analysis to ensure that collisions are not being displaced to surrounding roadways.
